# Self-Esteem and Body Image in Affective Psychotic Disorder with Adolescent Onset: A Clinical Analysis

**DOI:** 10.1192/j.eurpsy.2026.11198

**Published:** 2026-07-31

**Authors:** S. Pérez-Sánchez, C. Pérez-Sánchez, E. Gonzalez-Reigadas, I. Martín-Herrero

**Affiliations:** 1 Psychiatry; 2 Medicina Familia, Servicio Murciano de Salud, Murcia, Spain

## Abstract

**Introduction:**

In schizoaffective disorder, atypical antipsychotics are considered a first-line therapeutic option, often in combination with mood stabilizers. However, one of the most frequent adverse effects is weight gain, which may contribute to deterioration of body image and negatively affect self-esteem and psychosocial functioning.

**Objectives:**

1. To evaluate self-perception and psychosocial adjustment in patients with schizoaffective disorder with symptom onset during adolescence who are currently receiving oral brexpiprazole or aripiprazole.

To examine whether self-perception differs according to the prescribed treatment.

**Methods:**

A retrospective study was conducted including patients diagnosed with schizoaffective disorder of adolescent onset who attended outpatient mental health consultations over a 9-month period. A cross-sectional assessment was performed using the E-PICA scale. Statistical analysis was carried out with SPSS version 23.0.

**Results:**

Twenty-three patients were included: 60% women (mean age 38.6 years) and 40% men (mean age 40.5 years). Men sought psychiatric consultation earlier and received their diagnosis at a younger age compared to women (men: symptom onset at 16.8 years, diagnosis at 27.1; women: onset at 20.5 years, diagnosis at 37.1).

Brexpiprazole was prescribed in 54% of patients, with a similar distribution by sex (53% men; 55% women). Among those treated with aripiprazole, the following outcomes were observed: mild psychosocial impact in 18% (all women), moderate impact in 18%, and severe impact in 9%. In contrast, patients treated with brexpiprazole reported mild impact in 30% and moderate impact in 24% of cases, with no severe scores recorded.

**Conclusions:**

Female patients with schizoaffective disorder treated with brexpiprazole demonstrated better body image perception and self-esteem, with psychosocial impact rated as mild. Male patients showed greater impact, more frequently associated with oral treatment. Longitudinal follow-up and future studies are warranted to further explore associated factors and to compare outcomes with other therapeutic options.

**Disclosure of Interest:**

None Declared

